# Identification of QTL controlling meat quality traits in an F_2 _cross between two chicken lines selected for either low or high growth rate

**DOI:** 10.1186/1471-2164-8-155

**Published:** 2007-06-08

**Authors:** Javad Nadaf, Hélène Gilbert, Frédérique Pitel, Cécile M Berri, Katia Feve, Catherine Beaumont, Michel J Duclos, Alain Vignal, Tom E Porter, Jean Simon, Samuel E Aggrey, Larry A Cogburn, Elisabeth Le Bihan-Duval

**Affiliations:** 1 Station de Recherches Avicoles, INRA, Centre de Recherches de Tours, 37380 Nouzilly, France; 2 Station de Génétique Quantitative et Appliquée, INRA, Centre de Recherches de Jouy-en-Josas, 78352 Jouy-en-Josas, France; 3 Laboratoire de Génétique Cellulaire, INRA, Centre de Recherches de Toulouse, 31326 Castanet-Tolosan, France; 4 University of Maryland, Department of Animal & Avian Sciences, College Park, MD 20742, USA; 5 University of Georgia, Department of Poultry Science, Athens, GA 30602, USA; 6 University of Delaware, Department of Animal & Food Sciences, Newark, DE 19717, USA

## Abstract

**Background:**

Meat technological traits (i.e. meat pH, water retention and color) are important considerations for improving further processing of chicken meat. These quality traits were originally characterized in experimental lines selected for high (HG) and low (LG) growth. Presently, quantitative trait loci (QTL) for these traits were analyzed in an F_2 _population issued from the HG × LG cross. A total of 698 animals in 50 full-sib families were genotyped for 108 microsatellite markers covering 21 linkage groups.

**Results:**

The HG and LG birds exhibit large differences in body weight and abdominal fat content. Several meat quality traits [pH at 15 min post-slaughter (pH15) and ultimate pH (pHu), breast color-redness (BCo-R) and breast color-yellowness (BCo-Y)] were lower in HG chickens. In contrast, meat color-lightness (BCo-L) was higher in HG chickens, whereas meat drip loss (DL) was similar in both lines. HG birds were more active on the shackle line. Association analyses were performed using maximum-likelihood interval mapping in QTLMAP. Five genome-wide significant QTLs were revealed: two for pH15 on GGA1 and GGA2, one for DL on GGA1, one for BCo-R and one for BCo-Y both on GGA11. In addition, four suggestive QTLs were identified by QTLMAP for BCo-Y, pHu, pH15 and DL on GGA1, GGA4, GGA12 and GGA14, respectively. The QTL effects, averaged on heterozygous families, ranged from 12 to 31% of the phenotypic variance. Further analyses with QTLExpress confirmed the two genome-wide QTLs for meat color on GGA11, failed to identify the genome-wide QTL for pH15 on GGA2, and revealed only suggestive QTLs for pH15 and DL on GGA1. However, QTLExpress qualified the QTL for pHu on GGA4 as genome-wide.

**Conclusion:**

The present study identified genome-wide significant QTLs for all meat technological traits presently assessed in these chickens, except for meat lightness. This study highlights the effects of divergent selection for growth rate on some behavioral traits, muscle biochemistry and ultimately meat quality traits. Several QTL regions were identified that are worthy of further characterization. Some QTLs may in fact co-localize, suggesting pleiotropic effects for some chromosomal regions.

## Background

Meat-type chickens have been intensively selected for a long time mainly on growth rate, which has reduced the age at market weight (i.e. ~2 kg live body weight). Selection efforts have improved body composition (i.e. increasing breast yield and lowering carcass fatness). However, these improvements have also led to indirect and sometimes deleterious effects on meat quality traits (i.e. pH, color and water holding capacity) [1]. The importance of these traits has increased following the development of further processing of chicken meat. Post-mortem pH appears to be a key factor in meat quality, since low pH increases the risk of producing pale meat with reduced water holding capacity, which affects the quality of further processed products [2]. On the other hand, results from a study of breast meat samples taken from a commercial processing plant suggests that dark meat with high pH has a shorter shelf-life [3]. Stress and behavioral activity on the pre-slaughter shackle line and muscle glycogen content at slaughter time have been shown to be involved in the variations of pH15 and pHu [4,5]. High heritability (h^2^) values were observed for several meat quality traits in chickens (h^2 ^= 0.35 to 0.57) slaughtered under controlled experimental conditions [6] and in turkeys (h^2 ^= 0.12 to 0.21) slaughtered under commercial conditions [7]. Therefore, these traits could be efficiently improved by phenotypic selection. However, only selection on collaterals could be applied, decreasing the efficiency and increasing the cost of such selection programs. So, identifying markers and genes associated with meat characteristics would be of great interest. Although QTL have already been reported in chicken for many traits, there are very few QTL reported for meat characteristics [8]T. To our knowledge, the suggestive QTLs for meat color on GGA2, GGA5 and GGA8 are the only ones reported for the chicken [9,10]. In the present study, we have compared meat quality traits from two experimental chicken lines divergently selected for growth rate (i.e. HG and LG lines) [11]. We have also presently characterized their behavioral response on the schackle line, using measurements as described by Debut et al. [4]. After checking that meat quality traits differed between the two lines, an F_2 _population was produced and used for QTL detection, with 108 microsatellite markers. The data were analyzed using QTLMap [12,13] and QTLExpress [14] softwares. QTLMAP makes no assumption either about fixation of the alleles in the founder lines or the number of the alleles segregating at QTL. As in our experiment, the founder lines may not be fixed for the QTLs alleles underlying meat quality traits, testing a corresponding free-assumption model is of special interest. However, in the case where we are dealing with a biallelic QTL fixed in the founder lines, using a simpler model such as the one underlying QTLExpress software may be relevant. The results obtained by this web-based software are also more comparable with those of other studies [8]. Several strong QTL controlling meat quality traits in chicken were revealed using both softwares.

## Results

### Characterization of meat quality traits in HG and LG founder lines as well as in their F2 cross

The phenotypic trait means and corresponding standard deviations for each founder line and their F2 cross are presented in Table 1. At 9 weeks of age, body weight (BW) was 2.8-fold higher (P < 0.001) in the HG than in the LG line. A large correlated response was also observed for abdominal fat which was about 12-fold higher (P < 0.001) in HG birds compared with the LG birds. A significant difference of lower magnitude was also observed for breast yield, which was higher (P < 0.001) in HG birds. Interestingly, potentially correlated responses to divergent selection were observed for breast meat traits as well as for growth rate. The color of breast meat was lighter (BCo-L) in HG chickens, which also exhibited lower redness (BCo-R) and yellowness (BCo-Y) in meat color. Post-mortem pH values also differed between the two lines, with HG birds exhibiting lower pH15 and ultimate pH values. Furthermore, HG chickens exhibited a higher breast muscle glycogen-equivalent content at death than LG chickens (98.5 ± 3.3 *μ*mol/g for HG vs.77.4 ± 3.0 *μ*mol/g and for LG line, n = 8, P < 0.01). Behavioral measurements on the pre-slaughter shackle line clearly indicated that LG birds were less active than HG birds. Indeed, the percentage of straightening up events (SU) was estimated at 18% in LG and 43% in HG (P ≤ 0.002). In addition, median values for the duration of wing flapping (WF) on the pre-slaughter shackle line were equal to 0 and 9 sec in LG and HG birds, respectively (P ≤ 0.0001). Indeed, quite significant negative phenotypic correlations were found between pH15 and SU or WF duration (-0.58 and -0.79 in HG line, respectively; -0.54 and -0.55 in LG line, respectively).

**Table 1:** Body weight, body composition and meat quality traits in high (HG) and low growth (LG) lines at 9 weeks of age (mean ± standard deviation)

	HG	LG	*P*-value	F2
*Chickens (n)*	53	56		698

*Growth and body composition*				
Body Weight (g)	1922 ± 157	683 ± 67	<.0001	1127 ± 185
Abdominal Fat	2.5 ± 0.7	0.2 ± 0.2	<.0001	1.6 ± 0.9
Breast Yield	11. 4 ± 0.8	10.4 ± 0.8	<.0001	11.4 ± 0.5

*Breast meat quality traits*				
Drip loss (%)	2.3 ± 1.2	2.1 ± 1.5	ns	1.2 ± 0.7
Lightness(BCo-L)	48.3 ± 3.2	45.6 ± 1.8	<.0001	47.3 ± 2.4
Redness (BCoR)	-0.2 ± 0.8	1.6 ± 0.7	<.0001	1.0 ± 0.9
Yellowness (BCo-Y)	9.4 ± 1.2	13.3 ± 1.4	<.0001	11.7 ± 1.5
pH 15 min (pH15)	6.20 ± 0.22	6.33 ± 0.16	0.0004	6.33 ± 0.18
Ultimate pH (pHu)	5.74 ± 0.09	6.14 ± 0.14	<.0001	6.01 ± 0.15

It was also of interest to compare phenotypic traits between the founder lines and the F2 progeny (Table 1), although data were not obtained at the same time. The average F2 traits were in the mid-range between the trait means of the founder lines, except for meat drip loss (DL) and to a lower extent pH15 and breast yield (BY). The phenotypic correlations between meat traits estimated from the F2 population were small to moderate. The most significant ones (p < 0.0001) were found between pH15 and DL (-0.33), BCo-Y and BCo-R (0.48) and between BCo-Y and pHu (-0.32).

### QTL analysis of meat quality traits using QTLMAP software

Table 2 shows the location and density of markers on the chicken chromosomes (GGA). Following QTLMAP analysis, estimates for five significant genome-wide and four suggestive QTLs are summarized in Table 3. The analyses applied to identify these QTLs included all families, but the number of heterozygous sire families varied from 1 to 5 according to the QTL (Table 4). The phenotypic variance explained by the QTL ranged from 12% for the QTL affecting DL on GGA1 to 31 % for the QTL associated with yellowness on GGA11 (Table 3). The study revealed QTLs for all traits under investigation except BCo-L. Highly significant QTLs were found for DL on GGA1, pH15 on GGA1 and GGA2, BCo-Y and BCo-R on GGA11. Suggestive QTLs were found for BCo-Y on GGA1, pHu on GGA4, pH15 on GGA12 and DL on GGA14 (Table 3). For the QTL controlling BCo-Y and BCo-R on GGA11 the alleles responsible for deeper coloring could be traced back to LG line for all sire families, except sire 1. Sire 1 exhibited no QTL effect for BCo-Y and one QTL effect for BCo-R but in the opposite direction (Table 4). The origin of the low allele for DL and high allele for pH15 on GGA1 was the HG line. While all sire families were informative for DL, only one sire was segregating for the QTL associated with pH15 on GGA1. The origin of the alleles for the other QTLs differed according to the families.

**Table 2:** Number of markers, map length, first and last markers for each chromosome (*GGA*)

*GGA*	Marker number	Map length (cM)	First marker	Last marker
1	17	541	MCW168	MCW108
2	17	468	MCW205	MCW157
3	11	287	ADL177	MCW037
4	6	231	ADL317	LEI073
5	8	166	LEI082	ADL298
6	5	115	LEI192	ADL323
7	6	125	LEI064	ADL315
8	3	67	MCW305	LEI136
9	3	84	LEI028	ADL132
10	4	65	ADL209	LEI112
11	3	51	MCW097	ADL308
12	3	62	ADL372	LEI099
13	3	37	MCW213	MCW110
14	2	25	ADL118	MCW123
15	3	55	ADL206	MCW211
17	2	32	ADL293	ADL199
18	2	17	ADL304	MCW217
19	2	13	MCW266	MCW256
26	3	41	ADL330	LEI074
27	3	39	MCW233	ADL376
28	2	6	ADL349	MCW227

**Table 3:** Estimation of QTL for meat quality traits in a novel F2 chicken population

		QTLMAP					QTLExpress		
		
Trait^1^	*GGA*	Location^2^	Flanking markers	LRP^3^	Var^4 ^(%)	*P*-Value (Chr)^5^	*P*_Value (Chr)^5^	F values	Location (cM)
BCo-Y	1	174 (168–182)	ADL188 – ADL150	102	20.5	0.02*^6^	ns	5.13	49
DL	1	357 (352–367)	MCW200 – LEI139	114	12	0.002**^7^	0.02*	10.1	560(454–565)
pH15	1	396 (386–403)	LEI139 – MCW283	126	29	<0.0005**	0.04*	8.97	369(324–404)
pH15	2	249 (243–274)	MCW063 – ADL257	124	14	<0.0005**	ns	2.36	302
pHu	4	189 (140–200)	ADL331 – MCW240	99	18	0.02*	<0.0005**	21.71	191(182–231)
BCo-Y	11	62 (56–69)	ADL210 – ADL308	153	31	<0.0005**	<0.0005**	53.42	62(51–69)
BCo-R	11	68 (58–69)	ADL210 – ADL308	115	19	<0.0005**	<0.0005**	24.83	65(41–69)
pH15	12	24 (16–30)	ADL372 – ADL044	105	21	0.005*	ns	4.43	0
DL	14	20 (20–44)	ADL118 – MCW123	98	13.5	0.003*	ns	0.49	45

^1^BCo-Y = yellowness; DL = drip loss, pH15 = pH 15 minutes post-mortem; pHu = ultimate pH; BCo-R = redness; ^2^Location and confidence interval in cM; ^3^Likelihood ratio ^4^Variance explained by the QTL (%) ^5^Chromosome-wide P-Value; ^6^* = Suggestive; ^7^** = Genome-wide 1%

**Table 4:** Segregation of QTL in each sire family and origin of alleles

			Origin of the dominant allele	
			
Trait^1^	Chr	Heterozygous sire families	HG line	LG line
BCo-Y	1	1-3-4	1	3-4
DL	1	1-2-3-4-5	-	1-2-3-4-5
pH15	1	5	5	-
pH15	2	1-2-3-4-5	1-4	2-3-5
pHu	4	1-2-3-4	2-3-4	1
BCo-Y	11	2-3-4-5	-	2-3-4-5
BCo-R	11	1-2-3-4-5	1	2-3-4-5
pH15	12	1-2-4	2	1,4
DL	14	1-2-3-4-5	1-4	2-3-5

^1^BCo-Y = yellowness; DL = drip loss; pH15 = pH 15 minutes post-mortem; pHu = ultimate pH; BCo-R = redness.

### QTL analysis of meat quality traits using QTLExpress software

QTLExpress analyses (Table 3) confirmed two out of the five significant genome-wide QTLs found by QTLMAP, those for BCo-Y and BCo-R on GGA11. In addition, the QTL for pHu on GGA4 was highly significant at the genome level with QTLExpress software, whereas it was only suggestive by QTLMAP. Genetic map positions of the three QTLs identified as genome-wide by QTLExpress, for BCo-R and BCo-Y on GGA11 and pHu on GGA4, were about the same as those estimated by QTLMAP. High alleles for these QTLs originated from the LG line for BCo-R and BCo-Y and from the HG line for pHu, which was consistent with the results by QTLMAP. QTLExpress also revealed two suggestive QTLs for DL and pH15 on GGA1. Confidence interval for the QTL affecting pH15 included the likeliest position estimated by QTLMAP, and the origin of the high allele was confirmed to be from the HG line. However, the confidence intervals estimated for DL by QTLExpress and QTLMAP did not overlap (Table 3); furthermore, the origin of the high allele was found to be different, (i.e. HG line with QTLExpress). Collectively, these results suggest two distinct QTL for DL on GGA1 at positions 357 and 560 cM.

## Discussion

HG and LG chicken lines, which were obtained by divergent selection within a population originally derived from meat-type lines, represent a unique resource for analyzing the genetic control of growth related traits and the underlying mechanisms. An F2 resource population was created by crossing the HG × LG lines [11] to obtain this goal. Several meat quality traits were measured and the F2 progeny were genotyped. We found several strong QTLs for meat quality traits, which are first discussed in the HG and LG founder lines.

### Phenotypic traits in the HG and LG founder lines

HG birds had paler breast meat, i.e. lower redness (BCo-R) and yellowness (BCo-Y) and higher lightness values. Paler meat has also been observed in chicken selected for both high body weight and breast muscle yield than in their unselected controls, which appears to be related to a reduction in muscle heme pigment (myoglobin) content in the heavy BW chicken lines [1]. Heme pigment content was not measured in the HG and LG chickens. Higher lightness values in HG line could also result from a lower pHu. This is consistent with the negative phenotypic relationship (-0.24) found between breast meat ultimate pH and lightness in the F2 population and in other studies reporting strong negative genetic correlations of -0.91 in the chicken [6] and of -0.53 in the turkey [7]. In chicken breast meat, the ultimate pH depends on muscle glycogen-equivalent content at death [5]. Indeed, the lower ultimate pH of HG breast meat coincides with higher glycogen content at death. We also observed a significant difference between lines for pH15 values, with HG birds exhibiting lower pH15 values. Muscle activity before slaughter could hasten post-mortem glycolysis and decrease muscle pH15 [5]. Behavioral observations of birds on the pre-slaughter shackle line clearly showed that HG birds exhibited higher struggle activity at slaughter. The comparison of HG and LG production and meat quality traits suggest that genetic selection for growth rate could change several bird characteristics, including behavior, muscle biochemistry and ultimately meat characteristics. Further research is needed to identify the mechanisms and genes underlying variations in the meat quality traits, which were identified by the present study.

### Comparison of QTLMAP and QTLExpress for analysis of meat trait QTL

Both QTLMAP [12,13] and QTLExpress [14] software were used for analysis of QTL for meat quality traits. No assumption about allele distribution was made with the QTLMAP method. Association analyses were performed for each F1 parent, and residual variances were estimated for each sire family using a heteroskedastic model, which could increase the power of the analysis when several QTLs govern a given trait [15]. In contrast, the QTLExpress method assumed that alternative QTL alleles were fixed in the founder lines, and only one substitution effect was estimated over all families. A good agreement was observed between the two methods, although the level of significance obtained by QTLMAP was generally higher. Indeed, both methods revealed strong genome-wide QTL for meat redness and yellowness on GGA11. This was consistent with the fact that QTL alleles were segregating in all but one of the five F1 sire families, and appeared to be almost fixed in the founder lines. The origin of the high alleles could be traced back to the LG line, which was consistent with the deeper coloring of meat observed in this line. The QTL effects for meat redness and yellowness were consistent with the high positive correlation between these two traits and the similar positions of the two QTLs. Hence, these two QTL are likely a single one affecting both traits. Further studies are required to determine if the protein(s) coded by gene(s) at this position exert(s) a unique graduated response or a pleiotropic effect on meat color. To our knowledge, only three suggestive QTLs for meat color, located on GGA2, GGA5 and GGA8, have been published for the chicken [9,10] None of these meat-color QTL presented on the Chicken QTL database [16], was found in the present study, which could be due to the different genetic background of the birds or to differences in the method used to measure meat color.

Three other genome-wide QTLs were identified by QTLMAP analysis for DL on GGA1 and for pH15 on GGA1 and GGA2. A QTL for DL on GGA1 was only suggestive using QTLExpress, with the high allele coming from the HG line. However, the origin of the high allele traced back to the LG line by QTLMAP. As discussed earlier, the two QTL identified by the different softwares at different positions (at 357 and 560 cM) could represent two distinct QTLs for which the alleles are mainly in a repulsive phase. In such cases, the effect of the QTL is usually underestimated [17]. This could explain why the effect of this QTL was the lowest one (12%) estimated by QTLMAP. The QTL for pH15 on GGA1 and GGA2, identified as genome-wide by QTLMAP, were respectively found as suggestive and non-significant by QTLExpress. Such a discrepancy between the two methods could be due to the small number of heterozygous sires (i.e. for the QTL on GGA1) and/or to changes in origin of the high allele according to sire families (i.e. for the QTL on GGA2). A similar feature was observed for two other suggestive QTLs identified by QTLMAP but not by QTLExpress (for yellowness on GGA1, pH15 on GGA12 and for DL on GGA14).

One QTL was found for pHu, which was identified as genome-wide by QTLExpress but only suggestive by QTLMAP. This was not expected because of the specificity of the two methods discussed above. Finally, it was surprising that no QTL was found for meat color lightness by either method of analysis, although the two founder lines were different in the BCo-L trait. However, the present genome scan only partially covered the complete chicken genome (about 2500 cM compared to the actual size of 4200 cM [18,19]); therefore, several QTL regions governing meat quality traits could have been missed.

The widely used "one LOD drop-off method" was applied to obtain 95 percent confidence intervals of the QTL [20]. This technique tends to under-estimate confidence interval sizes, and others [21] have suggested using a 2-LOD support interval to ensure reaching a 95 percent confidence interval. Some studies also showed that LOD-score-based confidence intervals were biased [22]. It should be noted that this method only provides a rough approximation of the confidence interval for locating a QTL. Thus, additional markers must be developed to refine the position of these meat quality QTL and to scan the regions of the genome not covered by the present study. Furthermore, new experimental designs such as recombinant progeny testing [23] or advanced intercross lines [24] could be used to refine the most interesting QTL regions.

## Conclusion

Several important QTLs for meat traits have been described in a novel F2 resource population created from an intercross of HG and LG lines. Some relationships between some behavioral traits and meat pH were observed. This is the first study to reveal significant QTL for meat quality traits in the chicken. Application of a statistical model that does not assume the number of alleles segregating for QTL and their origin in the founder lines appears to be a useful approach, as evidenced by obtaining a higher level of significance for the QTLs. Pleiotropic effects were also suggested for some chromosomal regions. This study indicates that divergent selection for growth rate has driven segregation of several meat quality traits. Once genes within chromosomal regions of interest have been precisely identified and the strength of QTL for meat quality determined, this could permit direct genetic selection through use of molecular markers, provided the same mechanisms are shared by commercial populations.

## Methods

### Phenotypic comparisons of HG and LG lines

The HG and LG lines were divergently selected for body weight (BW) at 8 and 36 weeks of age for more than 20 generations [11], resulting in a large difference in growth rate [25]. These founder lines and the F2 population from their intercross were used to assess effects of divergent selection on several meat quality traits and their genetic control.

For the present analysis of meat quality traits and muscle characteristics, 109 males (56 and 53 issued from HG and LG lines, respectively) were reared under standard management conditions and slaughtered at 9 weeks of age. Live body weight (BW), abdominal fat percentage and breast yield were measured in addition to meat pH at 15 min post-slaughter (pH15), ultimate pH (pHu), objective meat color at 24 h post-slaughter, and drip loss after 2 days of storage at 4°C, as described by Le Bihan-Duval et al. [6] (Hunterlab, Reston, VA 20190). The breast meat color (BCo) was measured using a Miniscan spectrocolorimeter with the CIE L*a*b* system, where L* is for the lightness (BCo-L), a* for the redness (BCo-R) and b* for the yellowness (BCo-Y). Higher L*, a* and b* values correspond to paler, redder and more yellow meat, respectively. The activity of the birds while on the pre-slaughter shackle line was also estimated by different measurements: straightening up (SU) of the body (head over the legs) was recorded during the period from the hanging to the electrical stunning and noted as a binary variable equal to 0 when the bird did not try to straighten up (absence) and to 1 otherwise (presence of straightening up). The total duration of wing flapping (TDWF) was recorded from hanging to electrical stunning. The breast muscle glycogen content at death was estimated on eight additional birds by line from measurement of the glycolytic potential [26] according to Berri et al[5].

### Phenotypic traits, genetic markers and F_2 _population

The F1 and F2 populations were issued from crossing of the HG and LG lines. Five F1 males (three F1 males issued from the cross of HG males by LG females, and two from LG males by HG females) were mated to 10 unrelated F1 dams each. A total of 698 F2 individuals originating from 50 full-sib families were produced in four successive hatches. At 9 weeks of age, body weight, abdominal fat percentage and breast muscle yield and meat traits [pH15, pHu, lightness (BCo-L), redness (BCo-R), yellowness (BCo-L) and DL] were measured on F2 birds as described earlier [6].

Genomic DNA was extracted from whole blood by phenol chloroform extraction. A total of 108 microsatellite markers covering 21 chromosomes, with an average distance of 23 cM between markers, were selected according to accessibility of the markers in the first genetic consensus map [18] and heterozygosity of the F1 parents. Fluorescently labeled microsatellite markers were analyzed on an ABI 3700 DNA sequencer (Applied Biosystems, Foster City, CA USA), and genotypes were determined using GeneScan Analysis 3.7 and Genotyper Analysis 3.7 software (Applied Biosystems, Foster City, CA USA). The GEMMA database was used to manage the informativity tests [27]. All F0, F1 and F2 (both males and females) animals were genotyped for all markers.

### QTL analysis software

Prior to QTL detection, the data were corrected for sex and hatch effects as estimated using PEST software [28]. QTL detection was performed first by using the QTLMAP software [12,13], based on interval mapping [20]. A maximum likelihood technique was applied to the mixture of full and half sib families, with no hypothesis concerning fixation of the QTL alleles in the founder lines. The QTL substitution effects (e.g. half the difference between QQ and qq genotypes) were estimated within each F1 family. The F1 haplotype probabilities were calculated from the marker information within the pedigree. Following assumptions of Mangin et al. [29], only the most probable sire genotype was considered and retained to compute the likelihood, whereas all dam genotypes with probability higher than 10% were considered. In practice, the likelihood could be linearized within sire families to improve computing efficiency.

F2 analyses using QTLExpress software were also performed [14]. Interval mapping was conducted using regression methods, in which alleles were assumed to be fixed in the founder lines. The founder line contrast was considered as identical in all the families. Haplotype transmission probabilities were computed with respect to approximations described by Haley and Knott [30].

### Significance thresholds

Three significance levels including chromosome-wide, genome-wide and suggestive were considered in this study. First, the chromosome-wide thresholds were derived empirically. When using QTLMAP, 2000 simulations under the null hypothesis of no QTL were performed [31] for each trait × linkage analysis group. When using QTLExpress, chromosome-wide thresholds were estimated from 2000 permutations, as suggested by Churchill and Doerge [32]. According to Lander and Kruglyak [33], the suggestive levels, for which one false positive is expected per genome analysis, were obtained for a specific chromosome as the contribution of that chromosome to the total genome length. The genome-wide thresholds were derived from chromosome-wide significance levels, using an approximate Bonferroni correction: *P*_*Genome*-*wide *_= 1 - (1 - *P*_*Chromosome*-*wide*_)^1/*r *^in which r was obtained by dividing the length of a specific chromosome by the length of the genome considered for QTL detection (2527 cM).

### Confidence interval and significance of the substitution effect in sire families

Following Lander and Botstein [20], 95% confidence intervals were set for QTL locations using the one-LOD drop-off method.

To test the significance of the sire effects estimated with QTLMAP, we modified the Lynch and Walsh equation [34], which describes the sample size (n) required to detect a completely additive QTL, located at the marker position and which explains a V_F _fraction of the total F2 phenotypic variance: n=1−VFVF(Z(1−[α/2])1−VF+Z(1−β))2
 MathType@MTEF@5@5@+=feaafiart1ev1aaatCvAUfKttLearuWrP9MDH5MBPbIqV92AaeXatLxBI9gBaebbnrfifHhDYfgasaacH8akY=wiFfYdH8Gipec8Eeeu0xXdbba9frFj0=OqFfea0dXdd9vqai=hGuQ8kuc9pgc9s8qqaq=dirpe0xb9q8qiLsFr0=vr0=vr0dc8meaabaqaciaacaGaaeqabaqabeGadaaakeaacqWGUbGBcqGH9aqpdaWcaaqaaiabigdaXiabgkHiTiabdAfawnaaBaaaleaacqWGgbGraeqaaaGcbaGaemOvay1aaSbaaSqaaiabdAeagbqabaaaaOWaaeWaaeaadaWcaaqaaiabdQfaAjabcIcaOiabigdaXiabgkHiTiabcUfaBHGaciab=f7aHjabc+caViabikdaYiabc2faDjabcMcaPaqaamaakaaabaGaeGymaeJaeyOeI0IaemOvay1aaSbaaSqaaiabdAeagbqabaaabeaaaaGccqGHRaWkcqWGAbGwcqGGOaakcqaIXaqmcqGHsislcqWFYoGycqGGPaqkaiaawIcacaGLPaaadaahaaWcbeqaaiabikdaYaaaaaa@4F3D@ where *α *is the risk of false positive detections and 1-*β *the power of QTL detection; for a given *α *and *β*, Z can be retrieved from statistical tables of standard normal distribution. In the present experiment, n = 140 in each sire family so, for a given *α *and *β *(in the present study *α *= 5% and *β *= 10%), we can infer a threshold value for V_F_, and test the significance of the substitution QTL effect estimated for each sire. Adjustments were applied to the equation, first to test only the sire allele effect (because QTLMAP makes no assumption about the number of QTL alleles and F1 dams can not be assumed to be heterozygous), and second to take into account the distance between marker and QTL (see appendix). Following this approach the critical value for the proportion of the family variance explained by the QTL (V_F_), ranged from 3.7% to 13.2%, depending on the distance between marker and QTL. As a consequence, only sires with a sufficient proportion of the family variance explained by the QTL were considered as heterozygous and included in the calculation of the average QTL substitution effect.

## Authors' contributions

JN carried out the QTL mapping analyses and drafted the manuscript. HG participated in the statistical analyses. FP and AV supervised the molecular aspects of the study and KF designed the set of microsatellite markers. ED supervised the study and carried out some statistical analyses. ED, CB, CMB, MJD, AV and JS participated in the design of the study, the data collection and helped to draft the manuscript. TEP, SEA and LAC participated in the design of the study and helped to draft and revise the manuscript. All authors read and approved the final manuscript.

## Appendix

### Significance of the substitution effect in sire families

Following Lynch and Walsh [34], consider a t-test for an F2 design, where the presence of a QTL is accepted if the difference between the means of the groups receiving the Q or q allele from the sire (M¯Q−−M¯q−
 MathType@MTEF@5@5@+=feaafiart1ev1aaatCvAUfKttLearuWrP9MDH5MBPbIqV92AaeXatLxBI9gBaebbnrfifHhDYfgasaacH8akY=wiFfYdH8Gipec8Eeeu0xXdbba9frFj0=OqFfea0dXdd9vqai=hGuQ8kuc9pgc9s8qqaq=dirpe0xb9q8qiLsFr0=vr0=vr0dc8meaabaqaciaacaGaaeqabaqabeGadaaakeaalmaanaaabaGaemyta0eaamaaBaaameaacqWGrbqudaWgaaqaaiabgkHiTaqabaaabeaaliabgkHiTmaanaaabaGaemyta0eaamaaBaaameaacqWGXbqCdaWgaaqaaiabgkHiTaqabaaabeaaaaa@3523@) is significantly different from zero. Supposing complete linkage between marker and QTL with additive value a: *E*(M¯Q−−M¯q−
 MathType@MTEF@5@5@+=feaafiart1ev1aaatCvAUfKttLearuWrP9MDH5MBPbIqV92AaeXatLxBI9gBaebbnrfifHhDYfgasaacH8akY=wiFfYdH8Gipec8Eeeu0xXdbba9frFj0=OqFfea0dXdd9vqai=hGuQ8kuc9pgc9s8qqaq=dirpe0xb9q8qiLsFr0=vr0=vr0dc8meaabaqaciaacaGaaeqabaqabeGadaaakeaalmaanaaabaGaemyta0eaamaaBaaameaacqWGrbqudaWgaaqaaiabgkHiTaqabaaabeaaliabgkHiTmaanaaabaGaemyta0eaamaaBaaameaacqWGXbqCdaWgaaqaaiabgkHiTaqabaaabeaaaaa@3523@) = *a*

Considering only half-sib families and assuming familial residual variance (σe2
 MathType@MTEF@5@5@+=feaafiart1ev1aaatCvAUfKttLearuWrP9MDH5MBPbIqV92AaeXatLxBI9gBaebbnrfifHhDYfgasaacH8akY=wiFfYdH8Gipec8Eeeu0xXdbba9frFj0=OqFfea0dXdd9vqai=hGuQ8kuc9pgc9s8qqaq=dirpe0xb9q8qiLsFr0=vr0=vr0dc8meaabaqaciaacaGaaeqabaqabeGadaaakeaaiiGacqWFdpWCdaqhaaWcbaGaemyzaugabaGaeGOmaidaaaaa@30E8@) independent from the sire QTL genotype:

σ(M¯Q−−M¯q−)2=σM¯Q−2+σM¯q−2=(1n1+1n2)σe2=4nσe2
 MathType@MTEF@5@5@+=feaafiart1ev1aaatCvAUfKttLearuWrP9MDH5MBPbIqV92AaeXatLxBI9gBaebbnrfifHhDYfgasaacH8akY=wiFfYdH8Gipec8Eeeu0xXdbba9frFj0=OqFfea0dXdd9vqai=hGuQ8kuc9pgc9s8qqaq=dirpe0xb9q8qiLsFr0=vr0=vr0dc8meaabaqaciaacaGaaeqabaqabeGadaaakeaaiiGacqWFdpWCdaqhaaWcbaGaeiikaGYaa0aaaeaacqWGnbqtaaWaaSbaaWqaaiabdgfarnaaBaaabaGaeyOeI0cabeaaaeqaaSGaeyOeI0Yaa0aaaeaacqWGnbqtaaWaaSbaaWqaaiabdghaXnaaBaaabaGaeyOeI0cabeaaaeqaaSGaeiykaKcabaGaeGOmaidaaOGaeyypa0Jae83Wdm3aa0baaSqaamaanaaabaGaemyta0eaamaaBaaameaacqWGrbqudaWgaaqaaiabgkHiTaqabaaabeaaaSqaaiabikdaYaaakiabgUcaRiab=n8aZnaaDaaaleaadaqdaaqaaiabd2eanbaadaWgaaadbaGaemyCae3aaSbaaeaacqGHsislaeqaaaqabaaaleaacqaIYaGmaaGccqGH9aqpdaqadaqaamaalaaabaGaeGymaedabaGaemOBa42aaSbaaSqaaiabigdaXaqabaaaaOGaey4kaSYaaSaaaeaacqaIXaqmaeaacqWGUbGBdaWgaaWcbaGaeGOmaidabeaaaaaakiaawIcacaGLPaaacqWFdpWCdaqhaaWcbaGaemyzaugabaGaeGOmaidaaOGaeyypa0ZaaSaaaeaacqaI0aanaeaacqWGUbGBaaGae83Wdm3aa0baaSqaaiabdwgaLbqaaiabikdaYaaaaaa@5F6C@, where n_1 _and n_2 _are the expected number of Q_ and q_ individuals and equal to n/2.

Assuming that n is large enough to consider a normal distribution for observed difference in means of the two groups, and denoting the fraction of the phenotypic variance (σZ2
 MathType@MTEF@5@5@+=feaafiart1ev1aaatCvAUfKttLearuWrP9MDH5MBPbIqV92AaeXatLxBI9gBaebbnrfifHhDYfgasaacH8akY=wiFfYdH8Gipec8Eeeu0xXdbba9frFj0=OqFfea0dXdd9vqai=hGuQ8kuc9pgc9s8qqaq=dirpe0xb9q8qiLsFr0=vr0=vr0dc8meaabaqaciaacaGaaeqabaqabeGadaaakeaaiiGacqWFdpWCdaqhaaWcbaGaemOwaOfabaGaeGOmaidaaaaa@30D2@) due to the segregation at QTL as V_F_:

. Then, following the notation of [35],

n=1−VF2VF(Z(1−[α/2])1−VF+Z(1−β))2
 MathType@MTEF@5@5@+=feaafiart1ev1aaatCvAUfKttLearuWrP9MDH5MBPbIqV92AaeXatLxBI9gBaebbnrfifHhDYfgasaacH8akY=wiFfYdH8Gipec8Eeeu0xXdbba9frFj0=OqFfea0dXdd9vqai=hGuQ8kuc9pgc9s8qqaq=dirpe0xb9q8qiLsFr0=vr0=vr0dc8meaabaqaciaacaGaaeqabaqabeGadaaakeaacqWGUbGBcqGH9aqpdaWcaaqaaiabigdaXiabgkHiTiabdAfawnaaBaaaleaacqWGgbGraeqaaaGcbaGaeGOmaiJaemOvay1aaSbaaSqaaiabdAeagbqabaaaaOWaaeWaaeaadaWcaaqaaiabdQfaAjabcIcaOiabigdaXiabgkHiTiabcUfaBHGaciab=f7aHjabc+caViabikdaYiabc2faDjabcMcaPaqaamaakaaabaGaeGymaeJaeyOeI0IaemOvay1aaSbaaSqaaiabdAeagbqabaaabeaaaaGccqGHRaWkcqWGAbGwcqGGOaakcqaIXaqmcqGHsislcqWFYoGycqGGPaqkaiaawIcacaGLPaaadaahaaWcbeqaaiabikdaYaaaaaa@502F@, where *α *and *β *are respectively the probabilities of detecting a false QTL and missing a true QTL.

Substituting the corresponding standard normal Z values for *α *= 5% and 1-*β *= 90%: n=1−VF2VF(1.961−VF+1.28)2
 MathType@MTEF@5@5@+=feaafiart1ev1aaatCvAUfKttLearuWrP9MDH5MBPbIqV92AaeXatLxBI9gBaebbnrfifHhDYfgasaacH8akY=wiFfYdH8Gipec8Eeeu0xXdbba9frFj0=OqFfea0dXdd9vqai=hGuQ8kuc9pgc9s8qqaq=dirpe0xb9q8qiLsFr0=vr0=vr0dc8meaabaqaciaacaGaaeqabaqabeGadaaakeaacqWGUbGBcqGH9aqpdaWcaaqaaiabigdaXiabgkHiTiabdAfawnaaBaaaleaacqWGgbGraeqaaaGcbaGaeGOmaiJaemOvay1aaSbaaSqaaiabdAeagbqabaaaaOWaaeWaaeaadaWcaaqaaiabigdaXiabc6caUiabiMda5iabiAda2aqaamaakaaabaGaeGymaeJaeyOeI0IaemOvay1aaSbaaSqaaiabdAeagbqabaaabeaaaaGccqGHRaWkcqaIXaqmcqGGUaGlcqaIYaGmcqaI4aaoaiaawIcacaGLPaaadaahaaWcbeqaaiabikdaYaaaaaa@468F@. Finally, to take into account the distance between marker and QTL (m), the sample size needed for an F2 population is approximated as nm=n(1−2c)2
 MathType@MTEF@5@5@+=feaafiart1ev1aaatCvAUfKttLearuWrP9MDH5MBPbIqV92AaeXatLxBI9gBaebbnrfifHhDYfgasaacH8akY=wiFfYdH8Gipec8Eeeu0xXdbba9frFj0=OqFfea0dXdd9vqai=hGuQ8kuc9pgc9s8qqaq=dirpe0xb9q8qiLsFr0=vr0=vr0dc8meaabaqaciaacaGaaeqabaqabeGadaaakeaacqWGUbGBdaWgaaWcbaGaemyBa0gabeaakiabg2da9maalaaabaGaemOBa4gabaGaeiikaGIaeGymaeJaeyOeI0IaeGOmaiJaem4yamMaeiykaKYaaWbaaSqabeaacqaIYaGmaaaaaaaa@3914@[34], where c is the recombination frequency, and by applying Haldane's mapping function c=1−e−2m2
 MathType@MTEF@5@5@+=feaafiart1ev1aaatCvAUfKttLearuWrP9MDH5MBPbIqV92AaeXatLxBI9gBaebbnrfifHhDYfgasaacH8akY=wiFfYdH8Gipec8Eeeu0xXdbba9frFj0=OqFfea0dXdd9vqai=hGuQ8kuc9pgc9s8qqaq=dirpe0xb9q8qiLsFr0=vr0=vr0dc8meaabaqaciaacaGaaeqabaqabeGadaaakeaacqWGJbWycqGH9aqpdaWcaaqaaiabigdaXiabgkHiTiabdwgaLnaaCaaaleqabaGaeyOeI0IaeGOmaiJaemyBa0gaaaGcbaGaeGOmaidaaaaa@36AC@[36], where m is the distance between QTL and the nearest marker, expressed in Morgan (M) units.
